# Feedback and feedforward methods in medical and higher health education: A scoping review

**DOI:** 10.1371/journal.pone.0354742

**Published:** 2026-07-30

**Authors:** Jill Vögelin, Irene Koenig, Iris Sterkele, Theresa Himmelsbach, Angela Blasimann, Evert Zinzen, Slavko Rogan

**Affiliations:** 1 Bern University of Applied Sciences, School of Health Professions, Bern, Switzerland; 2 Vrije Universiteit Brussel, Faculty of Physical Education and Physiotherapy, Brussels, Belgium; Federal University of Technology - Parana, BRAZIL

## Abstract

**Background:**

Programmatic assessment (PA) in health professions education positions continuous feedback and feedforward as key mechanisms for competence development. Feedback focuses on prior performance, whereas feedforward provides future-oriented guidance that supports agency and self-regulated learning. Although their educational value is widely acknowledged, implementation remains challenging and often emphasises performance outcomes rather than action and reflection.

**Methods:**

A scoping review was conducted to address the research question: “How are feedback and feedforward methods operationalised within assessment situations in medical and higher health professions education? The ECLIPSE framework (Expectation, Client group, Location, Impact, Professionals, Service) guided question formulation and searches in Embase, ERIC and PubMed. Articles published from 2015 up to February 2026 were included. Data were synthesized using descriptive and thematic synthesis. The methodological quality of the included studies was assessed using Joanna Briggs Institute (JBI) critical appraisal tools.

**Results:**

Database searches identified 3723 records. After deduplication and screening, 91 full texts were assessed, of which 14 studies met inclusion criteria. All studies involved medical students. Five methodological families were identified: expert feedback, self-feedback, peer feedback, video-based feedback, and feedforward. Expert and video-based approaches were consistently associated with performance gains, whereas self- and peer feedback supported reflection and learner agency but showed variable outcomes. Feedforward was rarely explicitly described, although several interventions incorporated forward-looking elements targeting performance.

**Conclusion:**

Current assessment practices rely on a limited set of recurring methods, with feedforward, predominantly focused on performance, often remaining implicit. Making feedforward explicit and actionable within assessment cycles might strengthen competence development, promote learner agency, and support continuous improvement in PA context.

## Introduction

Programmatic Assessment (PA) has gained increasing prominence in medical and health professions education as a comprehensive, learner‑centred approach that supports the longitudinal development of professional and interprofessional competencies, while fostering learner agency and responsibility for learning [1,2]. At its core, PA aims to shift assessment practices away from isolated, high-stakes examinations toward an integrated, longitudinal system in which assessment and learning are closely intertwined [3,4]. Within this framework, assessment is reframed from a primarily judgment-oriented activity into a process that supports learning through iterative feedback, feedforward and reflection.

Feedback plays a central role in this longitudinal system and is conceptualised not merely as information about past performance, but as a key developmental mechanism that facilitates reflection, promotes metacognition, and supports continuous improvement within a constructivist learning paradigm [5,6]. Rather than classifying competence at a single point in time, assessment information in PA is used to support learners’ sense-making, decision-making, and professional development over time [7,8].

Traditional feedback practices often emphasise behavioural correction and the identification of strengths and weaknesses. While such approaches may effectively shape immediate performance, they risk reinforcing deficit-oriented perspectives that can undermine learners’ autonomy, motivation and self-efficacy, particularly when attention is primarily directed toward performance gaps [9,10]. In contrast, contemporary constructivist approaches to feedback emphasise learner agency, dialog engagement, and the co‑construction of meaning. These approaches prioritise the development of evaluative judgement and position feedback as a collaborative process aimed at supporting improvement rather than solely evaluation [6]. Such principles align closely with PA, which seeks to create psychologically safe learning environments that enable iterative development and sustained competency growth [1].

In recent years, feedforward has emerged as a complementary concept that extends the developmental function of feedback. These approaches prioritise the development of evaluative judgement and position feedback as a collaborative process aimed at supporting improvement rather than solely evaluation [11]. This future-oriented perspective reflects socio-constructivist assumptions of learning as an active, iterative process of participation, reflection, and meaning-making [11,12]. Within PA, such forward-looking processes are particularly important, as progression decisions rely on the interpretation of longitudinal assessment information.

Despite increasing recognition of the importance of feedback and feedforward, their effective integration into assessment practices in health professions education remains challenging. Existing literature demonstrates considerable variation in definitions, conceptualisations, and practical applications, particularly in competency-based and authentic assessment contexts [13,14]. Although feedforward is widely advocated, its operationalisation in educational practice remains insufficiently specified. In particular, there is a lack of structured overviews that systematically describe how feedback and feedforward methods are used within assessment situations and how they align with contemporary learning theories and PA principles.

Given this conceptual complexity and methodological heterogeneity, a scoping review was conducted to map existing literature and provide an overview of feedback and feedforward methods in medical and health professions education [15].

This scoping review aimed to examine how feedback and feedforward are operationalized within assessment situations, to identify prevailing and emerging approaches, and to provide a structured overview that can inform the development of assessment practices within programmatic and competency-based frameworks.

Research question

How are feedback and feedforward methods operationalised within assessment situations in medical and higher health professions education?

## Methods

### Study design

The review follows the Preferred Reporting Items for Systematic Reviews and Meta-Analyses extension for Scoping Reviews guidelines (PRISMA-ScR) [15]. This ensures transparency, reproducibility, and methodological rigor across all stages of the review, including eligibility criteria, search strategy, data charting, and synthesis.

### Protocol and registration

A protocol was preregistered at PROSPERO with the following number: CRD420251112360. A protocol was also registered at OSF with the following registration https://doi.org/10.17605/OSF.IO/G6SYA.

### Eligibility criteria

This scoping review considers qualitative, quantitative, and mixed methods articles in English and German language in which feedback and feedforward methods were evaluated in assessment situations in higher health professions education. Any article with assessment methods in workplace based clinical settings was excluded.

### Information sources

The literature search was conducted in the electronic databases Embase, ERIC (via Ovid), and PubMed. All databases were searched for studies published between January 2015 and February 2026. The final search was conducted in February 2026.

### Search

**Fig 1:**
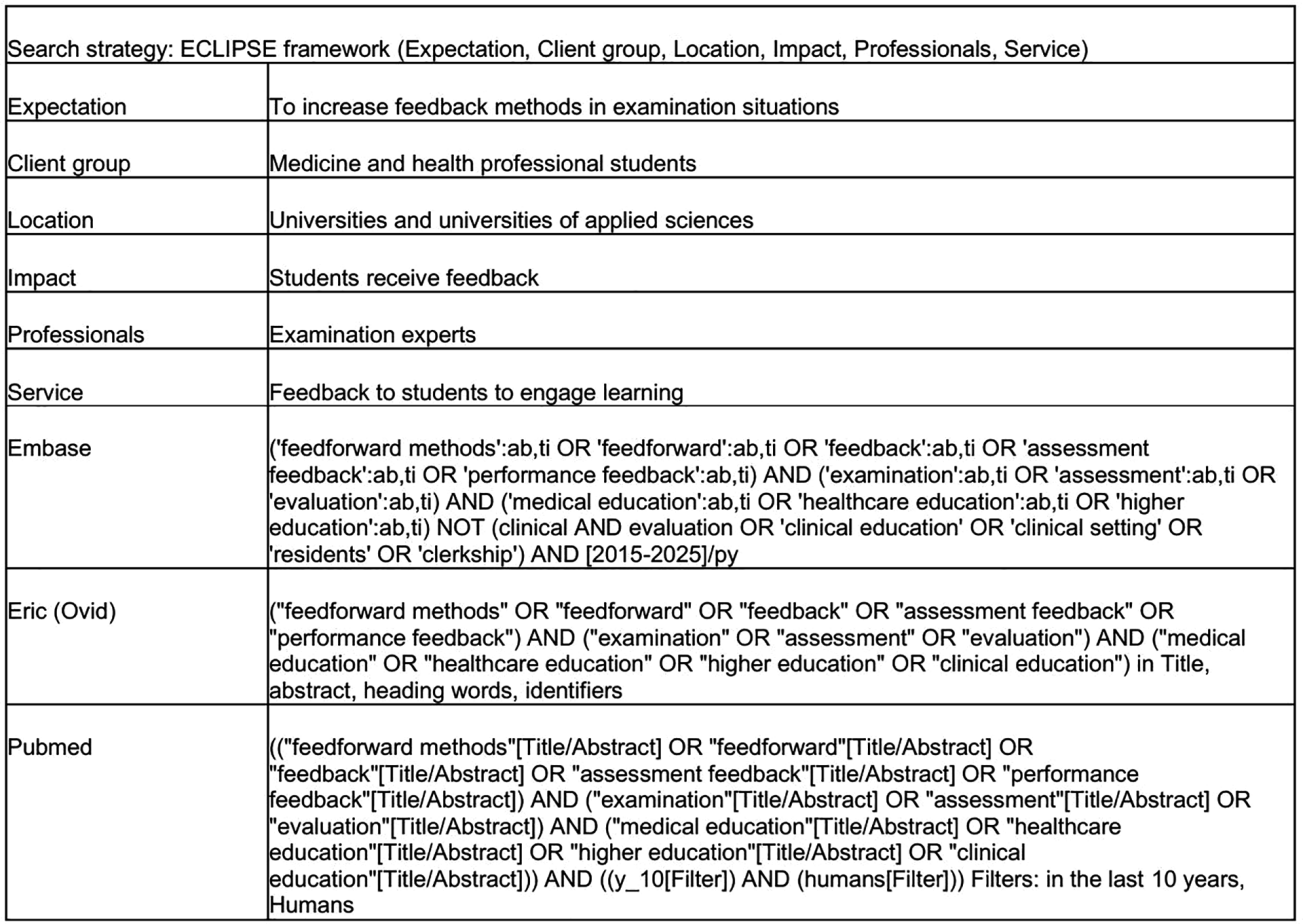
**Search strategy literature search.** ECPLIPSE framework and search string for Embase, Eric (Ovid) and Pubmed..

The ECLIPSE framework (Expectation, Client group, Location, Impact, Professionals, Service) was used to formulate the research question and guide the identification of relevant search terms [16]. Articles published from 2015 onwards were included. Although the conceptual foundations of feedforward and learner-centered feedback predate this period, the mid-2010s represent a phase of consolidation and increasing prominence of these concepts in literature. This period is characterized by a stronger emphasis on learner agency, dialogic feedback processes, and future-oriented approaches, alongside a notable rise in related publications and integrative frameworks [11,17]. The time restriction was therefore applied pragmatically to capture contemporary conceptualisations (Fig. 1).

### Selection of sources of evidence

Study selection followed a two-stage screening process. First, two reviewers independently screened titles and abstracts against predefined eligibility criteria. This process was supported by Rayyan, a web-based tool designed to facilitate collaborative screening in systematic and scoping reviews [18]. Studies identified as potentially eligible by either reviewer were advanced to full-text review.

**Fig 2:**
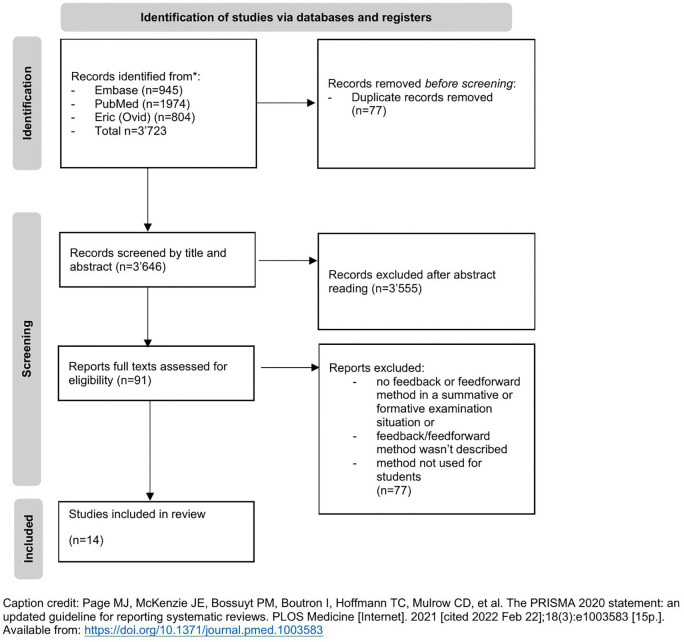
PRISMA 2020 flow diagram for systematic reviews. Study selection process and identification of studies via databases and registers.

In the second stage, at least two reviewers independently assessed the full-text articles for inclusion. Discrepancies at both the title/abstract and full-text screening stages were resolved through discussion and consensus, and, if necessary, through consultation with a third reviewer. The study selection process is presented in the PRISMA flow diagram (Fig 2)

### Data charting process

**Fig 3:**
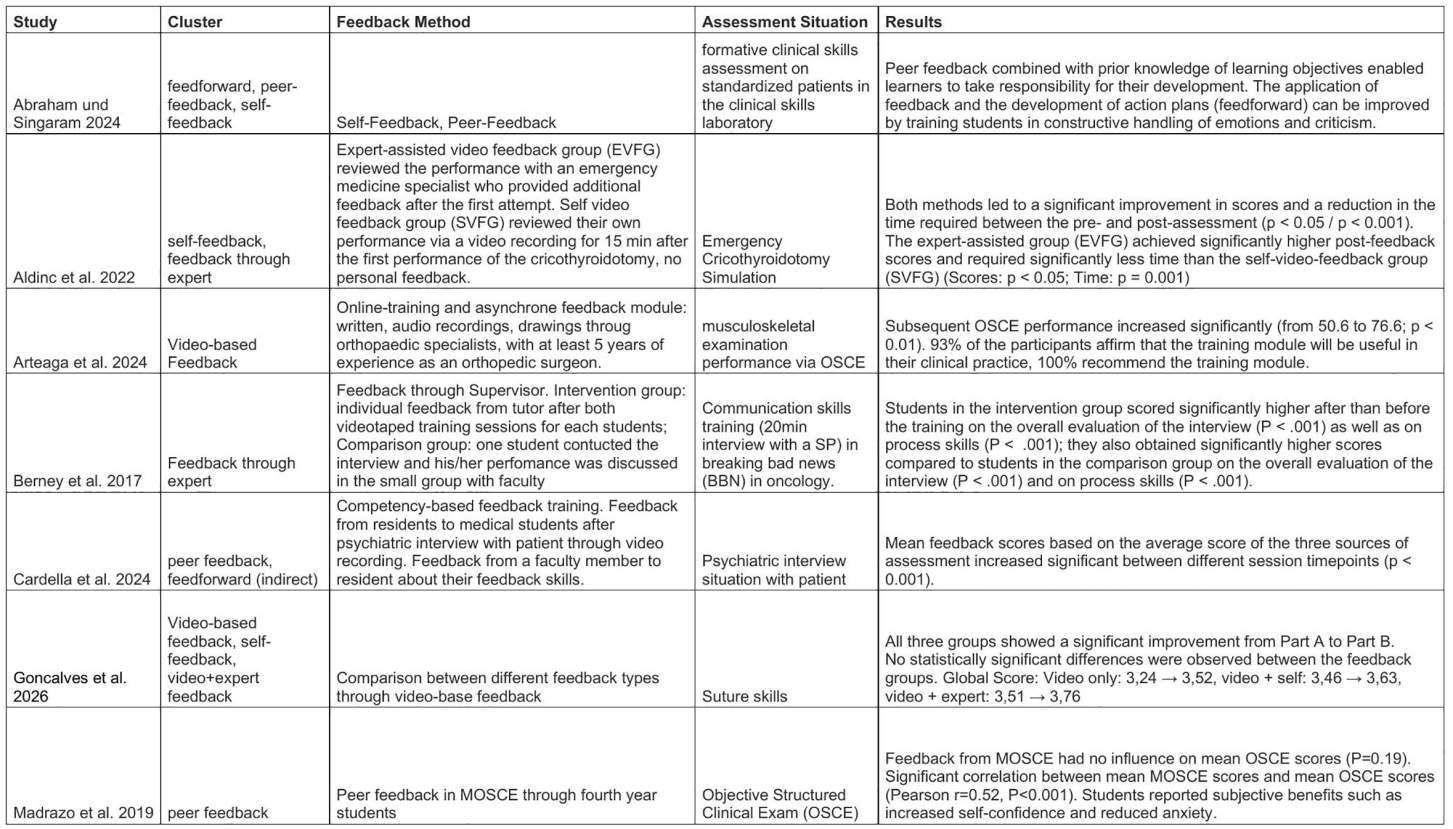
Data extraction. Data extraction part I.

**Fig 4:**
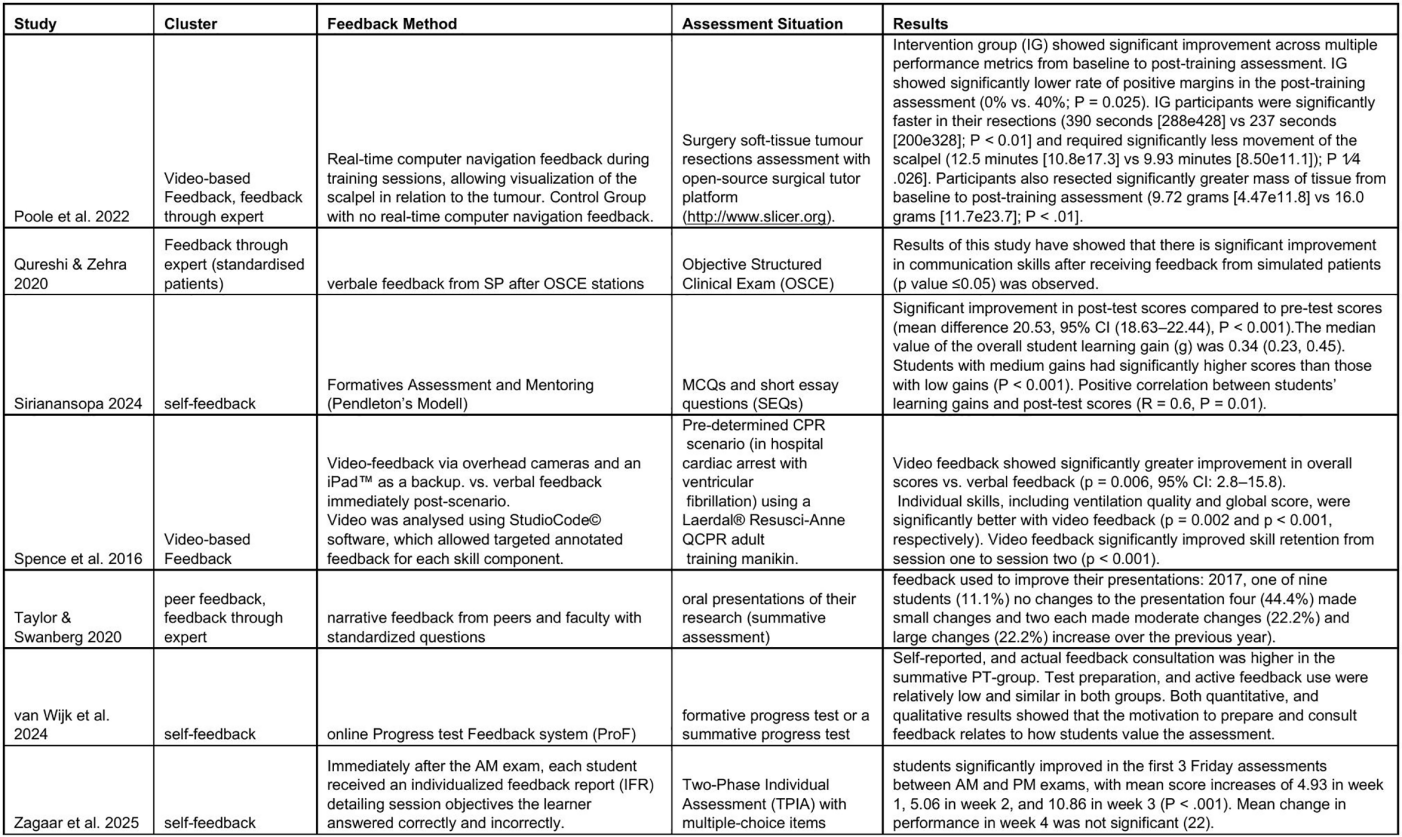
Data extraction. Data extraction part II.

The data extracted from the studies were conducted by at least two independent authors individually. A Microsoft Excel spreadsheet (Microsoft® Excel® for Microsoft 365 MSO (Version 2402 Build 16.0.17328.20346), 64 Bit) was used to extract the following study characteristics: article title, author’s name, year, study design, country of origin, study population, aim, setting, feedback and types of feedback/feedforward methods. Allocation and indexing were reviewed by a second author, and any disagreements were discussed until a consensus was reached. To support thematic analysis, feedback and feedforward strategies were further categorized into subgroups based on their characteristics, purpose, and implementation context. The spreadsheet containing the data extraction is shown in Figs 3 and 4.

### Data items

The following data items were extracted from each included study: bibliographic details (authors, year of publication), study characteristics (study design, country of origin, study population, setting), and study aim.

In addition, variables related to feedback and feedforward were systematically extracted. These included the type of feedback provider (e.g., expert, peer, self), the method family (e.g., video-based feedback, narrative feedback, structured feedback approaches), and the delivery mode (e.g., one-to-one interaction, asynchronous video feedback, written formats).

Further variables captured the timing of feedback (e.g., immediate, delayed, between sessions, concurrent), and the presence and type of feedforward (e.g., explicit, implicit, none).

Outcome-related data were also extracted, including the outcome domain (e.g., procedural skills, communication, critical thinking, learning behavior) and the reported effect of the intervention (e.g., improvement, no effect), including direction and statistical significance where reported.

To support comparative analysis, feedback and feedforward methods were grouped into broader categories based on shared characteristics (e.g., expert feedback, peer feedback, self-feedback, video-based feedback), reflecting their role in the learning process and their implementation context.

Where studies did not explicitly define feedback or feedforward approaches, classifications were derived from the methodological descriptions provided by the authors. Any ambiguities were discussed among the reviewers to ensure consistency.

### Critical appraisal

**Fig 5:**
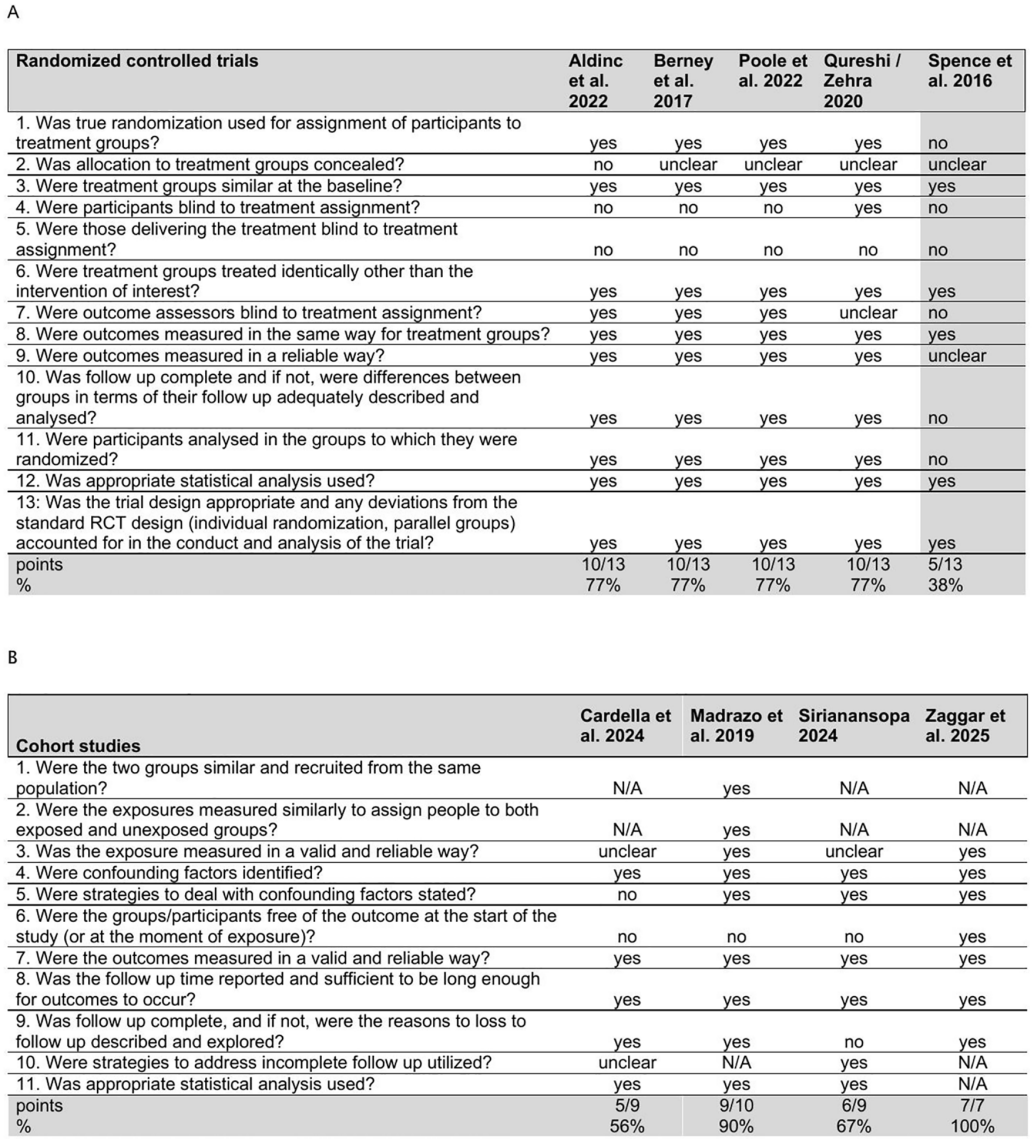
JBI Critical Appraisal Checklist. (A) JBI Critical Appraisal Checklist for randomized controlled trials. (B) JBI Critical Appraisal Checklist for cohort studies.

**Fig 6:**
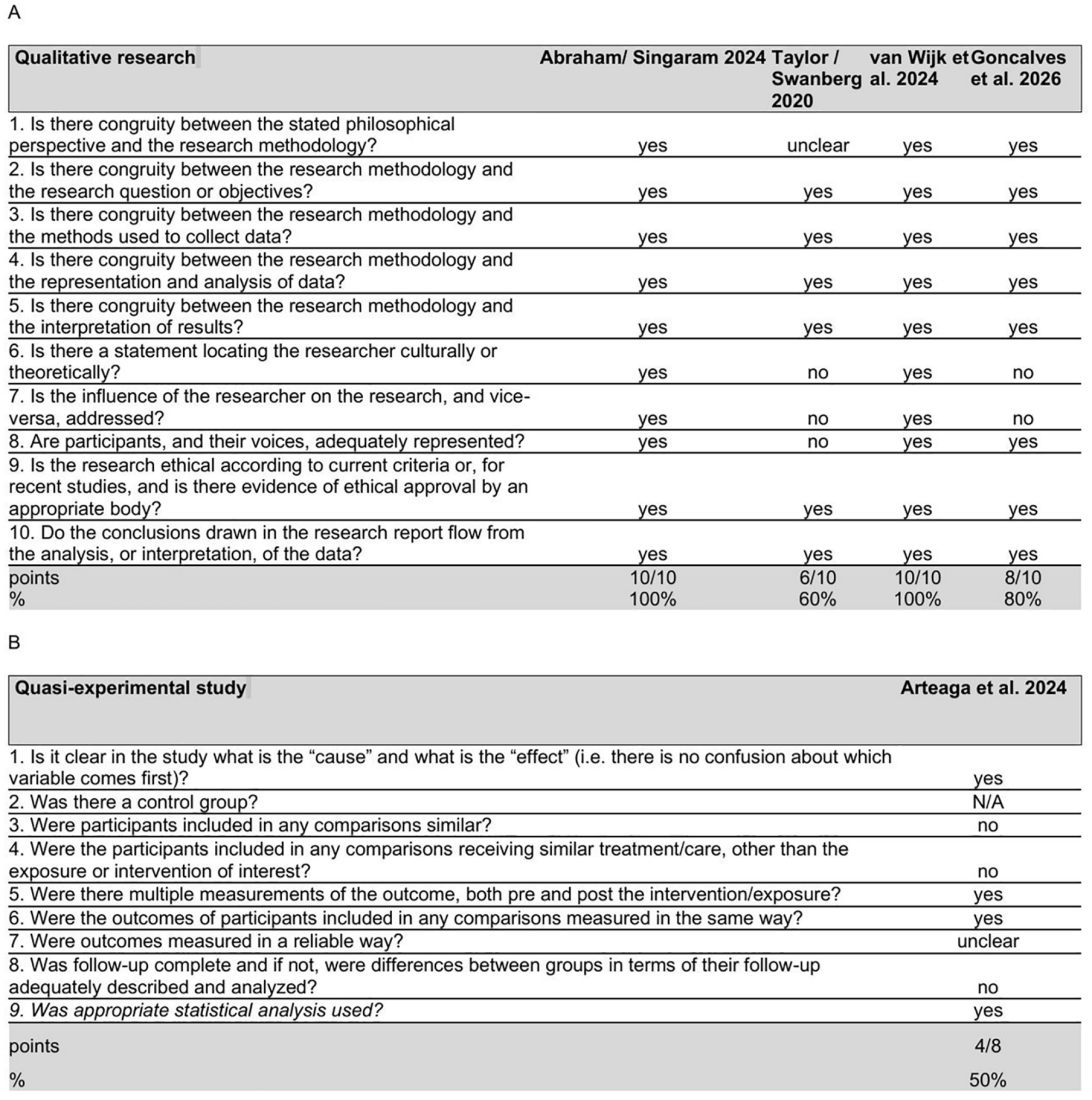
JBI Critical Appraisal Checklist. (A) JBI Critical Appraisal Checklist for qualitative research. (B) JBI Critical Appraisal Checklist for quasi-experimental studies.

The methodological quality of the included articles was independently assessed by three reviewers (JV, IS, TH) using the appropriate Joanna Briggs Institute (JBI) Critical Appraisal Tools, selected according to study design (Figs 5 and 6).

A critical appraisal was conducted with an overview of the methodological characteristics and potential limitations of the included evidence. In line with the scoping review methodology, studies were not excluded based on their methodological quality.

Any disagreements regarding study designs classification or appraisal outcomes were resolved through discussions and, where necessary, consulting with a fourth reviewer. The results of the critical appraisal were used to inform the interpretation of findings but were not used as a basis for study exclusion.

### Synthesis of results

Charted data were analyzed using a descriptive and thematic synthesis approach. First, key study characteristics and extracted variables were summarized descriptively to provide an overview of the included studies.

Subsequently, feedback and feedforward methods were analyzed across studies to identify recurring patterns and variations. Methods were grouped into broader categories based on shared characteristics, including feedback provider (e.g., expert, peer, self), delivery mode (e.g., verbal, written, video-based), and timing (e.g., immediate, delayed, or longitudinal).

A thematic analysis was then conducted to explore how feedback and feedforward were conceptualized and operationalized within assessment contexts. This included examining the presence and role of feedforward (e.g., explicit, implicit), as well as the intended function of feedback approaches (e.g., performance improvement, reflection, self-regulation).

Outcome domains and reported effects were synthesized descriptively, focusing on the direction and nature of reported outcomes rather than quantitative aggregation, given the heterogeneity of study designs and measures.

The synthesis aimed to map the range, characteristics, and implementation of feedback and feedforward methods rather than to evaluate their effectiveness.

### Ethics and dissemination

Due to the nature of scoping review and its secondary data analysis, ethical review is not required. The results of this review will be disseminated through a peer-reviewed publication to ensure the rigor and credibility of the findings.

## Results

### Selection of sources of evidence

A total of 3723 records were identified through database searching, (Embase: n = 945; PubMed: n = 1974; ERIC: n = 804). After removal of duplicates (n = 77), 3646 records remained for title and abstract screening.

Of these, 3555 records were excluded, resulting in 91 articles assessed for full-text eligibility. Following full text review, 77 articles were excluded for the following reasons: absence of feedback or feedforward method in formative or summative assessment contexts, lack of a clear description of the feedback/feedforward method, or inapplicability to student populations. In total 14 articles were included in the final analysis. The study selection process is illustrated in the PRISMA flow diagram (Fig 2).

### Characteristics of sources of evidence

All fourteen included studies examined cohorts of medical students (n = 14) [19–32], and only a single study incorporated an additional subgroup of psychiatric residents in medical students (n = 1) [23].

Five articles were designed as randomized controlled trials [20,22,25,26,29]. Four articles were designed as cohort studies [23,24,28,32], four articles as qualitative studies (including mixed methods and explorative qualitative designs) [19,27,30,31] and one experimental study [21].The present review included articles from Canada (n = 2), Chile (n = 1), Ireland (n = 1), the Netherlands (n = 1), Pakistan (n = 1), Portugal (n = 1), South Africa (n = 1), Switzerland (n = 1), Thailand (n = 1), Turkey (n = 1), and the USA (n = 3) [19–32].

### Critical appraisal within sources of evidence

Based on the JBI critical appraisal tools, 13 of the 14 included articles demonstrated moderate to high methodological quality (50–100%) [19–28,30–32], whereas one article scored below 50% [29]. Among four qualitative studies, three demonstrated very high methodological quality [19,27,31], while one article showed average quality [30].

Of the five randomized controlled trials (RCT), four were rated as methodologically robust [20,22,25,26], whereas one RCT was rated as poor [29]. Across all RCTs, blinding of participants or assessors was not feasible, as treatment allocation could not be concealed.

In contrast, the quasi-experimental study [21] was rated as average, mainly due to the lack of comparison of participants’ comparison groups and the absence of follow-up measures.

Among the four cohort studies, two were rated as very good [24,32], while two demonstrated average quality [23,28], primarily due to missing follow up data or because the outcome of interest was already known at baseline. Critical appraisal is available in Figs 5 and 6.

### Results of individual source of evidence

A total of 14 studies were included in the review [19–32]. Across these studies, five methodological families of feedback and feedforward approaches were identified: expert feedback, self-feedback, peer feedback, video-based feedback, and feedforward. These approaches were applied in assessment contexts within medical and health professions education, including Objective Structured Clinical Examinations (OSCEs), simulation-based training, and classroom-based assessments.

**Fig 7:**
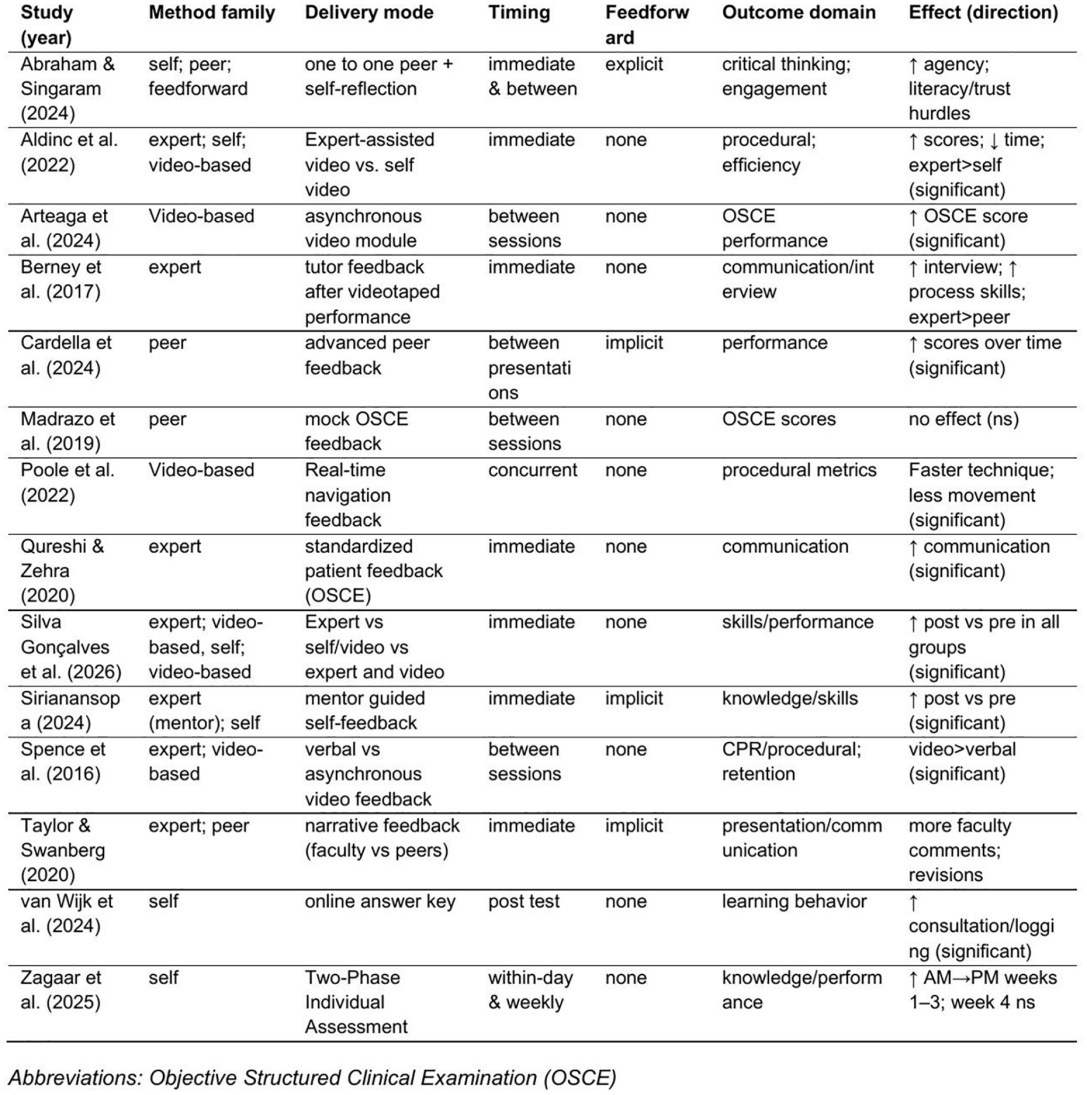
Methodological family groups. Key characteristics of included studies.

Key characteristics of the included studies, such as method family, delivery mode, timing, presence of feedforward, outcome domains, and reported effects, are summarized and illustrated in Fig. 7.

Most studies investigated procedural and communication outcomes, as well as learning behavior and engagement. The majority of studies reported positive effects of feedback interventions on performance and learning outcomes, although variability across methods and contexts was observed. A small number of studies, particularly in the context of peer feedback, reported no statistically significant effects [24].

### Synthesis of results

Five methodological families of feedback and feedforward approaches were identified: expert feedback, self-feedback, peer feedback, video-based feedback, and feedforward.

#### Expert feedback.

Expert feedback, provided by faculty members, clinicians, standardized patients, or mentors, was the most frequently reported approach and was typically structured and criterion-based [20,22,26–30].

Across studies, expert feedback was consistently associated with improvements in performance, efficiency, and communication skills. In comparative designs, expert-assisted approaches generally outperformed self-directed methods, particularly in short-term performance outcomes such as task completion time and post-test scores [20,22]. Mentor-supported approaches additionally promoted reflective processes and forward planning, even in the absence of direct performance ratings [28]. Where verbal expert feedback was contrasted with video-based formats, video-supported feedback demonstrated superior outcomes [29],

Overall, expert feedback appears to provide detailed, domain-specific guidance that supports both performance improvement and cognitive engagement.

#### Self-feedback.

Self-feedback centred on structured self‑reflection, often supported by artefacts (video review, online answer keys) or mentoring prompts [19,20,27,28,31,32].

These approaches were consistently associated with performance improvements, although typically to a lesser extent than expert feedback [20,27]. Lower-performing students appeared to experience greater challenges, particularly in relation to feedback literacy and confidence, and benefitted from additional scaffolding such as explicit criteria, exemplars, or guided reflection prompts [19,28].

In summative assessment contexts, increased engagement with self-feedback tools, such as more frequent consultation of answer keys, suggests stronger goal orientation under higher-stakes conditions [31].

#### Peer feedback.

Peer feedback ranged from informal, immediate one-to-one exchanges to structured feedback provided by trained or advanced peers [19,23,24,30].

The effectiveness of peer feedback appeared to be moderated by factors such as perceived credibility, trust, and learners’ feedback literacy. While some studies demonstrated significant improvements over repeated assessment points [23], others found no significant effects on objective performance measures such as OSCE scores [24].

Qualitative findings suggested that lower-performing students were more likely to question the credibility of peer feedback and engage less actively, whereas higher-performing students demonstrated greater engagement and confidence in peer feedback processes [19].

#### Video-based feedback.

Video-based feedback approaches included both asynchronous review and real-time feedback systems integrated into procedural training [21,25,29].

These approaches supported detailed observation, repeated viewing, and segmentation of performance, allowing for more precise analysis. All included studies reported significant improvements in procedural and communication outcomes. In comparative studies, asynchronous video feedback outperformed verbal feedback, while real-time systems improved efficiency by reducing unnecessary movements and enhancing task execution [25,29].

Video-based feedback thus appears particularly effective for skill acquisition and performance refinement in clinical training contexts [21,25,29].

#### Feedforward (explicit vs. implicit).

Feedforward was explicitly described in only one study [19], while several others incorporated implicit forward-looking elements [23,28,30]. Explicit feedforward involved clearly defined next steps, including goals, strategies, criteria, and timelines for future performance, often accompanied by an emphasis on learner accountability and ownership [19]. In contrast, implicit feedforward occurred when learners were encouraged to apply feedback to subsequent performance without structured guidance or clearly defined action plans.

The majority of studies did not explicitly include or describe feedforward components [20–22,24–27,29,31,32].

## Discussion

This review examined which feedback and feedforward methods are currently used in assessment situations in medical and higher health professions education, and how these methods are operationalized. The findings show that feedback and feedforward are operationalized through a combination of feedback sources (expert, peer, self), delivery modes (e.g., one-to-one, video-based, asynchronous), timing (e.g., immediate, between sessions, longitudinal), and varying degrees of structure, ranging from criterion-referenced expert guidance to learner-driven reflective approaches. Feedforward is operationalized either explicitly through structured action planning or implicitly as forward-looking guidance embedded within feedback processes.

Across fourteen studies, five methodological family groups were identified: expert feedback, peer feedback, self-feedback, video-based feedback, and feedforward. Expert feedback was most common, followed by peer and self-feedback and video-based approaches. Feedforward was rarely explicitly labelled, although several interventions contained a forward-looking logic aimed at guiding subsequent performance [23,28,30]. These patterns are consistent with PA as a development-oriented system that connects multiple assessment moments to support longitudinal learning than isolated judgements [1,2].

All feedback modalities were associated with short-term improvements in performance. However, studies involving experts input tended to report larger gains and greater efficiency compared to purely self-directed conditions, particularly when feedback was structured and criterion referenced or where mentors scaffolded reflection and planning [20,26–28]. Video-based approaches supported replay, segmentation, and focus analysis of performance Asynchronous video feedback outperformed verbal feedback in several studies, and real-time feedback systems improved efficiency by increasing speed and reduced unnecessary movement [25,29]. Online practice supported by focused feedback was associated with substantial improvements in objective structured clinical examination performance [21].

In contrast, the effects of peer and self-feedback were more variable and appeared to depend on such as credibility, trust and feedback literacy. Students performing lower reported more reservations and engaged less actively with peer feedback, whereas students performing higher engaged more confidently. These patterns highlight the importance of structured scaffolding and clear criteria, and support for feedback literacy [19,30]. Similarly, in progress testing, students engaged more frequently with self-feedback tools in summative contexts, suggesting that assessment design influences how feedback is used [31].

A key central conceptual clarification concerns the relationship between feedback and feedforward. Although often treated as distinct constructs, they are closely interconnected in practice. Feedforward rarely occurs in isolation but typically builds on reflection stimulated by feedback or self-assessment. The distinction lies primarily in temporal and functional orientation: feedback focuses on past performance, while feedforward directs attention toward future action by specifying what to do next, how, and under which conditions improvement can occur. This explains why several studies incorporated forward-looking elements without explicitly using the term [9,11,19]. The findings therefore suggest that feedforward should be considered a deliberate design feature rather than an implicit by-product of feedback processes.

From a constructivist perspective, a key feature of the current evidence is its strong focus on observable performance outcomes. Many interventions conceptualise feedback as a response to measurable indicators such as scores, time, or error rates. While these approaches align with assessment traditions and provide clear evidence of improvement, they may underrepresent processes such as sense-making, identity development, and evaluative judgement. Constructivist perspectives instead conceptualise feedback as a dialogic process that supports meaning-making, agency, and self-regulated learning [5,6,17]. The findings therefore do not suggest replacing behaviourist approaches with constructive models but rather integrating sequentially. Structured, criterion-referenced guidance may provide initial for orientation and calibration, followed by deliberate for reflection, self-regulation and learner ownership. This integration allows feedforward to become explicit and actionable within iterative learning cycle [19,30].

Reflection processes observed in the included studies aligned with experiential learning cycles. Video-based review supports reflection on concrete experience, mentor-guided discussion facilitates conceptual understanding, and subsequent practice enables active experimentation. This sequence may help explain the consistent effectiveness of video-based approaches and the added value of expert-supported feedback for rapid calibration [20,21,29].

Embedding feedback and feedforward in assessment design requires alignment at two levels. Content-focused learning goals ensure that feedback targets relevant knowledge and skills, while competence-oriented learning outcomes support integration into clinical reasoning, communication and professional performance [1,13]. Practically, feedback interactions should in a structured plan that specifies goals, strategies, criteria and timeframes, aligning the guiding questions “where am I going”, “how am I going”, and “where to next” [9].

Finally, an epistemic perspective helps explain why quantitatively oriented approaches often demonstrate strong effects. Positivist measurement frameworks privilege indicators such as speed, accuracy, and scores, which are readily quantifiable. While these outcomes are important, they may not fully capture deeper dimensions of learning. Mixed-methods approaches that integrate quantitative outcomes with qualitative analysis of learning processes may therefore provide a more comprehensive understanding of how feedback and feedforward support competence development [5,6].

### Limitation

Several limitations should be considered when interpreting these findings. The evidence base was concentrated in medical education. With one exception that included a subgroup of medical psychiatry students, all studies sampled medical students, which constrains transferability to other health professions programs. Notably, only 14 studies were included out of 3,723 identified records. While this reflects stringent eligibility criteria and systematic screening procedures, it raises concerns about the comprehensiveness and representativeness of the evidence base. This substantial reduction suggests conceptual ambiguity in how feedforward is operationalized or reported in the literature and may also indicate that relevant practices are underrepresented or insufficiently captured by current terminology and indexing.

Feedforward was rarely defined or operationalized explicitly and was often embedded implicitly within broader feedback processes, thereby reducing conceptual clarity and limiting precise attribution of mechanisms. This mirrors the wider literature that treats feedforward variably and underscores the need for early, actionable guidance between tasks [11]. Outcome measures were predominantly performance-based indicators such as scores, time, and error reduction. While useful, this behaviorist leaning may underrepresent constructivist aims such as sense making, agency, evaluative judgement, and identity development, which are central to contemporary feedback theory and to the alchemy of learning in authentic settings [5,6]. Primary studies also exhibited methodological constraints common to educational research. In the randomized controlled trials, blinding of participants or assessors was not feasible because allocation to visible pedagogical conditions could not be concealed [25,29]. Several non-randomized designs lacked comparison groups or did not include follow-up, and reporting was at times incomplete, which may bias effect estimates and can lead to conservative quality ratings despite structured appraisal tools [21,23,28]. Search and selection boundaries may have limited completeness: the review drew on Embase, ERIC, and PubMed, covered 2015 to February 2026, and included publications in English and German. Therefore, relevant studies in other databases, time frames, or languages may have been missed, and publication bias cannot be ruled out.

## Conclusion

To answer the research question, assessment situations in medical and higher health professions education deploy expert, peer, and self-feedback, as well as video-based approaches and both implicit and explicit forms of feedforward. Expert and video-based approaches demonstrate robust short-term benefits, whereas peer and self-feedback processes are effective when supported by adequate scaffolding. Feedforward is frequently present as an underlying logic but is rarely explicitly named or operationalized.

A key practical implication for programmatic assessment is to make feedforward explicit and actionable within each assessment cycle. This enables learners to translate performance-related information into concrete next steps, aligned with both learning goals and competence-oriented outcomes. Such processes require attention to credibility, trust, and psychological safety to ensure effective use.

Behaviorist and positivist approaches tend to demonstrate clear improvements, as they focus on measurable indicators such as speed, accuracy, and score. However, these measures do not fully capture deeper aspects of learning, including understanding, professional identity, and relational dimensions that are central to sustained and meaningful competence development.

Future research should provide more precise operationalization of feedforward, incorporate longer observation periods, and apply mixed-methods designs that include qualitative analyses of underlying mechanisms. Expanding sampling across health professions will further strengthen understanding of the independent and combined contributions of feedback and feedforward to competence development.

## Supporting information

S1 Checklist PRISMA 2020 Checklist. (DOCX)
